# Supplementary material for: Insights and Considerations in Development and Performance Evaluation of Generative Adversarial Networks (GANs): What Radiologists Need to Know
Source: Diagnostics (Basel). 2024 Aug 13;14(16):1756. doi: 10.3390/diagnostics14161756 (PMC11353572; doi:10.3390/diagnostics14161756)
Supplement: Supplementary file 1 [file diagnostics-14-01756-s001.zip › diagnostics-3163404-supplementary.pdf]

Supplementary material

I. CycleGAN – brain infarction images for augmentation

I.1 Background

Research on Cycle-GAN has been applied to a variety of medical fields, including brain image generation. Several studies have been conducted on T1-weighted image (T1WI), T2-weighted image (T2WI), and diffusion-weighted image (DWI). Van Nguyen et al. [65] conducted a study on tumor image generation from normal brain T2WI. They reported that the Fréchet inception distance (FID) score, which measures the similarity between real and generated images, was better than that of existing methods such as DCGAN and Tumor-GAN. A recent study used a switchable Cycle-GAN [60] to generate MRI sequences from T1WI and T2WI of normal brain images. The study converted T1WI to T2WI images and T2WI to T1WI. The study found that the structural similarity index measure (SSIM) and peak signal-to-noise ratio (PSNR) indicators were better than those of the existing Cycle-GAN, and the training time was more efficient.

Mizuki et al. [61] used a Cycle-GAN and U-net architecture to train a model on a relatively small dataset of DWI infarction images. This model was then used to generate synthetic images of patient with acute infarction patients. The results showed that the use of Cycle-GAN to regenerate images with high contrast improved the false positive rate by 1/3, and the Dice index from 0.47 to 0.55. However, the study did not investigate the image generation rate or qualitatively evaluate the image quality.

This study evaluated the generation rate of Cycle-GAN, which has not been previously studied in detail, for acute infarction lesions in DWI. We also evaluated the quality of the generated images based on the consistency rates of the radiologists.

I.2 Datasets

Employing brain DWI, this study retrospectively collected imaging data for a preliminary study from the PACS system of a tertiary university hospital. All patient names were collected anonymously. The period was from January 2019 to July 2021, and all brain images, including DWI images, were selected from the study description list. To obtain non-acute infarction images, they were searched for "No diffusion restriction" or "No acute infarction" in the conclusion, and acute infarction images were searched for "acute infarction." To minimize errors in Cycle-GAN data training, the exclusion criteria were as follows: 1) poor image quality due to severe artifacts, 2) infarctions with a small size or faint signal intensity, or 3) other combined lesions such as previous chronic infarction, hemorrhage, tumor, or severe brain atrophy.

Data collection was divided into a training dataset to train the Cycle-GAN and a test dataset to evaluate the performance. A total of 1885 patients with acute infarction were identified; 27 patients with poor image quality, 733 patients with infarction in two or more places, and 1,020 patients with other lesions, such as chronic infarction, hemorrhage, tumor, or severe brain atrophy, were excluded. In total, 205 patients with acute infarction were selected. A total of 1457 patients with no diffusion restriction were included, 22 patients with poor image quality, and 1,072 patients with other lesions were excluded. A total of 205 healthy patients were selected.

This study included 205 patients with acute infarctions, some of whom had multiple lesions. To account for this, we selected one DWI image per lesion, representing each lesion, for a total of 591 images. From the DWI images of 93 normal patients, we extracted 363 images from locations that were frequently observed in the acute infarction dataset. We performed left-right flip augmentation on each selected acute infarction and normal image and then doubled the number of images, resulting in a total of 1,182 and 726 images, respectively. Left–right flip augmentation was performed to increase the number of images and to prevent the learning of images that were biased toward the left or right. The test and training datasets were collected in the same manner. For acute and normal infarctions, we selected one image representing the lesion from each of the 15 patients (Table S1).

MRI scans were acquired using two 3T magnetic resonance (MR) systems (Achieva TX, Philips Medical Systems, Best, Netherlands, and 3T MAGNETOM VIDA, Siemens,

Erlangen, Germany). The imaging parameters for DWI were: repetition time =4458.1 ms, echo time = 60 ms; flip angle = 90°; slice thickness = 5 mm; pixel size = 0.9766 × 0.9766 mm; matrix size = 256 × 256; spacing between slice = 6 mm.

Table S1. The summary of Dataset for DWI (acute infarction, non-acute infarction)

	Training dataset		Test dataset		Total
	Acute infarction	Normal	Acute infarction	Normal	
No. of patient	203	93	15	15	326
No. of image slice	591	363	15	15	984
Age, mean ± SD, y	65.21 ± 14.08	66.34 ± 12.96	64.35 ± 16.12	62.63 ± 11.01	64.63 ± 14.55
Age, range	6-95	34-91	41-88	18-65	6-95
Sex					
Female	113	45	8	7	173
Male	92	48	7	8	155

II. pSp encoder combined styleGAN – brain vessel images for unsupervised anomaly detection

II.1 Background

Research on image generation has been conducted for sequences such as T1WI, T2WI, T1 enhancement, and DWI in brain imaging. However, research on TOF-MRA image generation, which represents vascular lesions, has been limited. Additionally, research on anomaly detection in vascular lesions has rarely been conducted [66]. The MIP image of TOF-MRA is difficult to segment and label even with deep learning, because the distribution, shape, and path of the blood vessels vary from patient to patient, and the number of anatomic variations varies from person to person. Additionally, brain vessels are characterized by the need to carefully examine small lesions during the evaluation by radiologists. Therefore, the generation of brain vessel images is difficult and requires high performance.

Most GAN models developed to date have not been able to generate brain MR images

with high enough resolution for medical applications [67]. To overcome this problem, recent studies have focused on combining various autoencoding systems with GANs [68]. Recently, active research has been conducted on style GANs that achieve high-resolution image quality. Usama et al. demonstrated the potential of style GANs for applications into other MR fields by generating high-quality brain tumor images [69].

This study proposes a model that generates the MIP of TOF-MRA images by combining a pixel2style2pixel (pSp) encoder and a Style-GAN which is a recently developed method for the generation of human faces. We expect a high-technique Style-based GAN model to effectively generate complicated blood vessel images. We also aim to apply this model for the detection of intracranial vessel anomalies.

II.2 Datasets

To train Style-GAN and to check whether the images were well generated, we collected an MIP dataset of TOF-MRA. This dataset was obtained from the PACS of a tertiary university hospital and the IXI dataset [70]. The IXI dataset contained publicly available adult brain images from multiple institutions. The dataset from the tertiary university hospital was retrospectively obtained from a study that included brain magnetic resonance angiography (MRA) data from May 2019 to July 2023, and was obtained under anonymous conditions. To select a patient group with normal brain MR angiography, we searched for “No steno-occlusive lesion nor aneurysm” or “Unremarkable MRA”. We excluded duplicate images of the same patient due to follow-up, coil or neck clipping procedures, and poor image quality due to in-saturation, motion, or metal artifacts. We included relatively common anatomical variations, such as hypoplastic A1, hypoplastic V4 segment and fetal posterior cerebral artery (PCA), without distinguishing them. A total of 753 patients were identified in the PACS without exclusion criteria, and 569 patients were selected from the IXI dataset. The test image was obtained from the PACS system of the tertiary university hospital in the same manner as the training dataset from 57 patients (Table S2).

MRA scans at the tertiary university hospital were acquired using two 3T MR systems (Achieva TX, Philips Medical Systems, Best, the Netherlands and 3T MAGNETOM VIDA, Siemens, Erlangen, Germany). The imaging parameters for MIP of TOF-MRA were as follows: repetition time = 20 ms, echo time = 25 ms; flip angle = 20°; slice thickness = 0.8 mm; matrix

size = 528 × 224; field of view = 200 × 200 mm^2. The MRA scans of the IXI dataset were collected from 600 healthy subjects at three hospitals in London. Imaging parameters of Hammersmith Hospital using a Philips 3T system were: repetition time = 17ms, echo time = 6ms; flip Angle = 16°; matrix size = 288 x 286; number of phase encoding steps = 286; echo train length = 0; Reconstruction Diameter = 240mm. The imaging parameters at Guy's Hospital using a Philips 1.5T system were as follows: repetition time = 20ms, echo time = 7ms; flip angle = 25°; number of phase encoding steps = 286; echo train length = 0; and Reconstruction Diameter = 240 mm. The imaging parameters of the Institute of Psychiatry using a GE 1.5T system are not available currently.

Table S2. The summary of Dataset for normal MIP of TOF-MRA

	Training dataset		Test dataset	Total
	PACS	IXI	PACS	
No. of dataset	653	569	57	1,279
No. of images	94,032	81,936	8,208	184,176
Age, mean ± SD, y	60.74 ± 11.98	49.49 ± 16.76	58.22 ± 16.12	55.60 ± 15.49
Age, range	19-92	19-86	18-85	18-92
Sex				
Female	374	314	33	721
Male	279	255	24	558

II.3 Quantitative Evaluation of the Performance

The image generation performance of the Style-GAN based generation model was evaluated using processed 8,208 test images from 57 patients with PACS data at the tertiary university hospital. All image processing and formation processes were performed using the same methods as those used for image training, and the images were generated in two ways; black background and color augmented images. A quantitative evaluation of how well the images were formed was performed using the Root Mean Square Error (RMSE), PSNR and SSIM. The evaluation of the three indicators was calculated as the average value of each of the 8,208 images generated from the black background and color augmentation images. The patients and rotation angles were subdivided, but the types of vessels were not. The average values of the black background and color augmentation images were compared. To determine whether there were any angles with significant differences in the three indicator values

depending on the angle, ANOVA was used. The Tukey-Kramer, a post-hoc test, was used to determine angles with significant differences.

II.5 Results of the Quantitative Performance

In the quantitative evaluation of the synthetic images from Style-GAN, the RMSE, PSNR, and SSIM values for the entire black background image were 16.57, 23.97, and 0.78, respectively. The RMSE, PSNR, and SSIM values for the color augmentation images were 23.73, 20.72, and 0.92, respectively. The average values of total RMSE and SSIM were higher in the color augmentation image, whereas the PSNR value was higher in the black background image (Table S3). The angles with a significant difference in values were similar between the RMSE, PSNR and SSIM. The vertical angle with the highest similarity to the input image was 300 degrees for PSNR and SSIM and the lowest vertical angle was 180 degrees for RMSE, PSNR and SSIM, regardless of the black background and color augmentation. There were no significant differences in the horizontal angles of the black background images. However, in the color augmentation images, the highest similarity to the input image was observed at 95 degrees for RSME, PSNR and SSIM, and the lowest similarity was observed at 250 degrees for PSNR and SSIM (Table S4).

Table S3. Quantitative evaluation and values of generated image of Style-GAN

	Black background	Color augmentation
RMSE (0, ∞)<br>mean ± SD	16.37 ± 2.77	23.73 ± 3.65
PSNR (0, ∞)<br>mean ± SD	23.93 ± 1.46	20.72 ± 1.31
SSIM (-1, 1)<br>mean ± SD	0.78 ± 0.07	0.92 ± 0.92

Table S4. The horizontal and vertical angles of the highest and lowest similarity in values of generated image of Style-GAN

		Black background			Color augmentation		
		RMSE	PSNR	SSIM	RMSE	PSNR	SSIM
Horizontal angle (degree)	Highest	70	-	-	95	95	95
	Lowest	-	-	-	65	250	250
Vertical angle (degree)	Highest	110	300	305	300	300	300
	Lowest	180	180	170	180	180	180

III. Limitation and Future study

The CycleGAN research has focused on brain infarction, but since there is already a significant amount of clinical imaging data available, augmentation in this area is less meaningful. However, if CycleGAN is applied to rare diseases, augmentation becomes highly valuable. In future studies, it can be applied to T1WI, T2WI, Susceptibility-weighted image (SWI), and T1 contrast enhancement (T1CE) sequences, which require higher resolution, and will be applied to the diagnosis of tumors, white matter diseases, and congenital anomalies.

In the pSp combined StyleGAN research, the blurring of the margins, diameter irregularity and ambiguity of course in small vessels are drawbacks that prevent it from providing image quality that is sufficient for clinical application. By developing preprocessing techniques to increase the amount of learning information or using a conditional GAN, these limitations might be addressed, potentially making it suitable for clinical use. The ultimate goal of our study is to propose an automated anomaly detection model for MIP of TOF-MRA in actual clinical settings. This will help to reduce the workload of radiologists, who are often understaffed, and shorten the diagnostic time and treatment time in emergency situations such as hemorrhage and acute infarction.
